# Multi-Objectives Optimization of Ventilation Controllers for Passive Cooling in Residential Buildings

**DOI:** 10.3390/s18041144

**Published:** 2018-04-09

**Authors:** Krzysztof Grygierek, Joanna Ferdyn-Grygierek

**Affiliations:** 1 Faculty of Civil Engineering, The Silesian University of Technology, Akademicka 5, 44-100 Gliwice, Poland; 2 Faculty of Energy and Environmental Engineering, The Silesian University of Technology, Konarskiego 18, 44-100 Gliwice, Poland; joanna.ferdyn-grygierek@polsl.pl

**Keywords:** optimization, genetic algorithm, fuzzy logic controller, detached house, passive cooling, energy demand

## Abstract

An inappropriate indoor climate, mostly indoor temperature, may cause occupants’ discomfort. There are a great number of air conditioning systems that make it possible to maintain the required thermal comfort. Their installation, however, involves high investment costs and high energy demand. The study analyses the possibilities of limiting too high a temperature in residential buildings using passive cooling by means of ventilation with ambient cool air. A fuzzy logic controller whose aim is to control mechanical ventilation has been proposed and optimized. In order to optimize the controller, the modified Multiobjective Evolutionary Algorithm, based on the Strength Pareto Evolutionary Algorithm, has been adopted. The optimization algorithm has been implemented in MATLAB^®^, which is coupled by MLE+ with EnergyPlus for performing dynamic co-simulation between the programs. The example of a single detached building shows that the occupants’ thermal comfort in a transitional climate may improve significantly owing to mechanical ventilation controlled by the suggested fuzzy logic controller. When the system is connected to the traditional cooling system, it may further bring about a decrease in cooling demand.

## 1. Introduction

Buildings account for about 40% of the global energy consumption [1,2,3] and contribute to over 40% of the total world CO_2_ emissions [4,5]. The largest contributors to this high energy consumption are heating, ventilation and air conditioning (HVAC) systems [6,7,8]. In residential buildings, cooling is also becoming more popular. While the heating demand can be effectively reduced by better thermal insulation of a building, cooling plays a more significant role in the overall energy demand of buildings [9]. Additionally, increased comfort expectations in summer time are contributing to the rise in cooling demand.

Mechanical cooling with electrically driven units is usually applied to achieve thermal comfort. Therefore, in developing countries, air-conditioning may account for more than half of the electricity consumption of a single dwelling [10]. Consequently, passive cooling solutions become more popular e.g., ventilative cooling methods. Ventilative cooling refers to the use of ventilation air in order to reduce or eliminate the need for mechanical cooling. Ventilative cooling can be applied through both mechanical (by fans) and natural (by openings and use of thermal gradients) ventilation strategies, as well as a combination of both [11]. Free cooling by ventilation is one of the most effective techniques for cooling since the ambient cool air is directly used to reduce the inside air temperature. The building’s structure is then used as a heat sink [12,13]. The increase in number of publications in recent years regarding the energy conservation in buildings through ventilative cooling illustrate its large energy saving potential in the near future. For example the work of Alonso et al. [11] examines the application of ventilative cooling in cold climates through simulations of an already existing kindergarten in Norway. Using simulation, the researches, compare the hybrid window ventilation system to DCV and VAV fan system (both without cooling) and only window controlled natural ventilation system. The results show important energy savings when using ventilative cooling as an outcome of the low outdoor temperatures and the same applies for night cooling. In the study by Inard et al. [12], the free cooling potential by ventilation for office buildings is evaluated by the free-running temperature. The free-running temperature approach is based on the energy balance of heat gains and losses. The approach was applied to 14 office rooms in a passively cooled office building in Germany and is used to estimate the potential and to evaluate the total energy saving by free cooling by ventilation. The paper by Yao et al. [14] presents an investigation of the natural ventilation cooling potential of office buildings in the five generally recognised climate zones in China using the thermal resistance ventilation model, which is a simplified, coupled, thermal and airflow one. The acceptable operative temperature for naturally conditioned space supplied by the ASHRAE Standard 55 has been used for the comfort temperature setting. In turn, the paper by Santamouris et al. [15] analyses energy data from above two hundred air conditioned residential buildings using night ventilation techniques in Greece. The relation of the cooling demand of the buildings with the specific contribution of night ventilation has been investigated. It has been found that night ventilation applied to residential buildings may decrease the cooling load up to 40 kWh/m^2^/year with an average contribution close to 12 kWh/m^2^/year.

The effectiveness of passive techniques depends directly on site conditions, varying not only from season to season but also along the daytime. Consequently, not every alternative might be a solution for a given location, but local climatic conditions must be carefully considered [16]. In the study by Artmann et al. [9] we can find an evaluation of the climatic potential for passive cooling of buildings by night-time ventilation in all climatic zones of Europe. The results show a high potential for night-time ventilative cooling over the whole of Northern Europe and still a significant potential in Central, Eastern and even some regions of Southern Europe. However, due to the inherent stochastic properties of weather patterns, a series of warmer nights can occur at some locations, where passive cooling by night-time ventilation alone might not be sufficient to guarantee thermal comfort.

When ventilation is used for cooling, variable airflow rates should be used in order to achieve comfortable room temperatures and to minimize the energy demand for ventilation. Thus, free-cooling, requires obviously the existence of a potential for cooling and needs control mechanisms for the airflow [12]. The paper by Rinaldi et al. [17] analyses the potential of building automation systems for ventilative cooling in residential buildings. In relation to internal and external temperature, an optimized control strategy of window opening is developed to ensure adequate levels of indoor thermal comfort, reducing energy consumption for cooling. The control of ventilation was optimized by a variable set-point using a particle swarm optimization method with objective function that minimizes the thermal discomfort hours. In turn, Castilla et al. [18] propose a multivariable nonlinear model predictive control system to maintain thermal comfort and indoor air quality by means of Heating, Ventilating, and Air Conditioning (HVAC) systems and natural ventilation. The main objective is to minimize the energy consumption necessary to achieve the comfort. In [19] the authors use an energy management algorithm implemented in the EnergyPlus simulation to control the natural ventilation. The control algorithm consists of indoor air quality based on CO_2_ sensors, thermal comfort to prevent the overcooling and the risk of air draft. Some researchers have investigated temperature as a suitable variable for controlling ventilation in homes. Homod and Sahari [20] developed a model to study the performance of natural and hybrid ventilation systems controlled by indoor temperatures and Predicited Mean Vote (PMV) in a single-family house in Kuala Lumpur, Malaysia. By turning off the air conditioning when it is not needed, 24 h cooling needs were reduced at least 8% in the cross-flow strategy and at least 28% in the optimized hybrid strategy.

In smart ventilating systems when using ambient air for ventilation, separate strategies to provide winter and summer ventilation airflow are needed. Winter ventilation, mainly for maintaining indoor air quality, usually needs minimal required airflow. Summer ventilation is mainly provided for thermal comfort and the flow rate required is larger compared to those needed for winter ventilation. The provision of summer ventilation is associated with the risk of overheating and has been identified as one of the critical factors for the application of natural ventilation by designers and users [21]. Thereby, to evaluate the applicability of any passive solution, a comprehensive analysis of the heating and cooling demand is needed. This requires a thorough study of the climate, as well as the provision of detailed information about the target building and about the indoor comfort expectations [22].

## 2. Research Problem Determination

Thermal comfort and occupant thermal satisfaction are very important for building designers. A poor indoor climate, especially indoor temperature, may contribute to the discomfort on the part of the occupants, and in some cases increase the risk of illness. Additionally, thermal comfort is a fundamental factor which influences the total energy demand of buildings as well as the cost of devices aimed at providing proper indoor air quality. Low outdoor temperatures in winter pose a major problem in Poland, where the climate is transitional. Every building designed for regular occupancy is equipped with adequate heating systems. Consequently, heating is of vital importance when it comes to total energy consumption. It is the purpose of research, therefore, to optimize the building partitions (thermal insulation of building envelope, the proper choice of windows) and heating systems in order to reduce heating demand [23,24]. In most cases the existing buildings lack any cooling systems and consequently too high temperatures in summer seasons create poor thermal conditions. However, the situation is changing and newly designed buildings are increasingly equipped with cooling systems. Air conditioning, which improves thermal comfort, is a costly investment. It is used in residential buildings in Poland only in summer months. Ventilation by open windows is another method used to improve thermal comfort in summer. Natural ventilation is hardy predictable and dependent on temperature difference and wind speed. It is relatively challenging to control such a system.

The main goal of this paper is to develop and test a control system for passive cooling based on mechanical ventilation using ambient cool air. It is a control system that will enable to provide the required minimum ventilation airflow in cold periods and to use the outdoor air for passive cooling of a building in transitional and hot periods. Such a solution is far more economical than mechanical air conditioning. A proper ventilation that uses ambient air to cool a building when the outdoor temperature is low can improve thermal conditions in a room during the warmer periods of the day, because the building’s structure can be used as a heat sink, which was mentioned in the literature review in the introduction.

The research in this field focuses mainly on office buildings [12], where night cooling is utilized. In the case of residential buildings occupied 24 h a day overcooling is not acceptable due to the comfort of residents. Yet, an adequate control of the outdoor airflow in such buildings may reduce cooling demand in mechanically ventilated and air-conditioned buildings or improve the thermal conditions (especially in warm periods) in the remaining buildings.

The paper explores possibilities for improving thermal comfort using mechanical ventilation in a typical residential building and transitional climate prevailing over Poland. A building was modelled using EnergyPlus and a new mechanical ventilation controller based on fuzzy logic theory was suggested for that purpose. The fuzzy logic controller was optimized using a genetic algorithm method. The modified version (in this study) of SPEA2_E/E_ method is used for multi-objective optimization of the basic parameters of the fuzzy logic controller (membership function and rule base). It was implemented in MATLAB. A new optimization procedure, which is based on the co-simulation of MATLAB and EnergyPlus programs is proposed. The analysis of the results shows it has been highly beneficial in terms of improving thermal conditions by the proper control of mechanical ventilation.

## 3. Method

### 3.1. Fuzzy Logic Controller—Description and Basic Assumption

A well-functioning mechanical ventilation ought to blow in the optimum values of air at appropriate times. In addition, this ventilation ought to improve thermal comfort without drastically increasing heating demand. Supplemental airflow to decrease indoor temperature in winter and in cool periods depends on the solar heat gains. These are, however, marginal cases, since heating is the main reason for energy consumption in this period in transitional climates. Thus the supplemental ventilation should be used for the high indoor temperature. When one turns on the supplemental ventilation at a temperature close to the heating set-point, the indoor temperature can drop considerably and then necessitate the use of supplemental heating. That will adversely affect the heating demand. Conversely, in summer when the indoor temperatures are high, in order to lower ones, supplemental outdoor airflow is used (it should run at the temperature determining the lowest limit of the required thermal comfort). It would be very unlikely to maintain the optimum temperature in a room without supplemental cooling, yet the number of hours without thermal comfort will decrease (particularly in the morning and evening). If, however, the outdoor temperature falls, keeping to the lower limit of permissible temperature may turn out to have an unfavorable outcome. Cooling may result in the room needing to be heated (even in the summer).

A low-speed supplemental ventilation airflow in winter and cool periods is enough to lower the room temperature. For summer it is the opposite. Additionally, the airflow ought to be calculated individually for every room.

**Figure 1:**
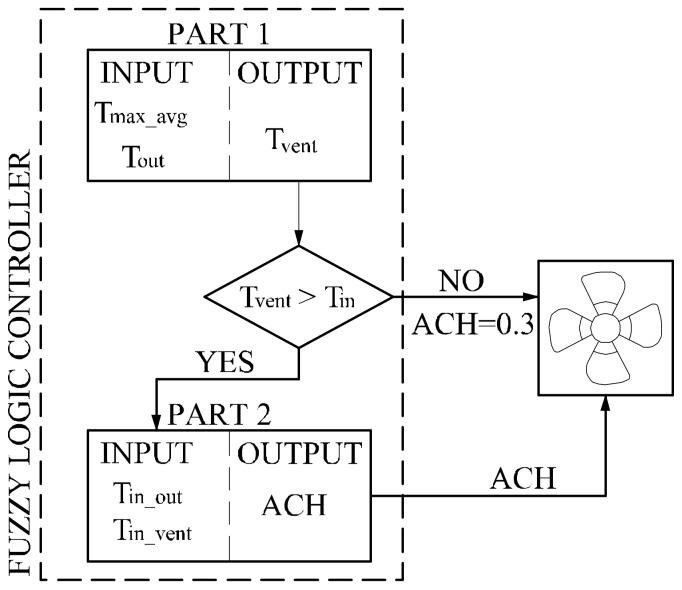
Framework of fuzzy logic control (FLC).

The above described conditions served as a basis for building a mechanical ventilation controller. Proportional-Integral-Derivative (PID) controllers are simple ones and are most widely used to control solutions for HVAC systems although it is often difficult to tune PID gains to their optimal values. In last few decades fuzzy logic has been widely used in literature to control HVAC systems [25]. The aim of fuzzy sets is mathematical representation of incomplete or imprecise information. Fuzzy logic controllers are more energy efficient, robust and have also a faster response to external disturbances because of their expert knowledge [26]. However, expert knowledge may not be enough to define all fuzzy logic controllers (membership function and rules). To solve this problem e.g., a self-learning fuzzy logic controller was developed in the work [27]. In our work a fuzzy logic controller is proposed to control ventilative cooling work. This controller was then optimized by genetic algorithms. A wide review of the literature related to the optimal HVAC system design can be found in the work by Ahmad et al. [26]. The new proposed fuzzy logic control (FLC) is divided into two parts (Figure 1). The outputs are the indoor air temperature (T_vent_) at which additional ventilation is activated and additional air change rate (ACH).

The following constraints are assumed in the FLC work:

- minimal air change rate (ACH) is 0.3 h^−1^,
- ventilation may be increased if the outside temperature (T_out_) is less than the inside room temperature (T_in_),
- additional ventilation will start if the room is not heated and the temperature exceeds the heating set point,
- input data for the FLC can only be temperature (indoor and outdoor air).

It was assumed that the input data for the calculation of output temperature (T_vent_—global factor for the whole building) are:

- weighted average of the highest outdoor temperatures (T_max_avg_) in the last three days,
- outdoor temperature at current time step (T_out_),
- and for the calculation of the additional instantaneous air change rate at each time step (ACH—local factor, calculated for each room separately) are:
- the temperature difference between indoor and outdoor temperature at the time step (dT_in_out_),
- the temperature difference between the indoor temperature and T_vent_ at the time step (dT_in_vent_).

It was assumed that input data are defined by the set of linguistic variables: low (L), medium (M) and high (H) and that output data are defined by the set of linguistic variables: very low (VL), low (L), medium (M), high (H) and very high (VH). In our work their shape as fuzzy rule base is created automatically and next is optimized in a genetic algorithm. The MIN-MAX method and center of gravity method were applied in the article to calculate output values.

### 3.2. Optimization of FLC

In recent years, a number of methods have been developed to generate and optimize fuzzy logic controllers (FLCs). Research concentrates mainly on two key components of an FLC system: tuning the membership function and learning the logic rules. Metaheuristic algorithms are able to find local optima, yet, they cannot guarantee an optimum solution. The most popular population-based algorithms are genetic algorithms. Genetic algorithms (GAs) are used for an adaptive heuristic search technique based on the process of natural selection [28]. They have been applied in the optimization of a wide range of issues [25,29]. Many studies use GAs for FLCs optimization [25,30]. GAs are suitable for global search the convergence rate, however is low. Particle swarm optimization (PSO) is the next method which was invented by Kennedy and Eberhart [31]. In recent years, they have become increasingly popular in optimization issues [32,33]. A wide review of works using PSO can be found in the article by Zhand et al. [34]. Increasingly, they are used in the optimization of FLCs [35,36]. Compared to GAs, PSO has a strong local search ability and weak global search ability. The hybridization of both algorithms speeds up the search process. This was used in the optimization of an HVAC system in Kusiak et al. [37] and for optimization of a fuzzy logic controller [38]. To simplify the optimization algorithm, only a GA is used for a multi-objective optimization of the FLC in our work. Research, where GAs are used to tune membership function (MF) and learn the logic rules, can generally be divided into four categories [39]: (1) tune MFs under a given set of logic rules; (2) select the logic rules with known MFs; (3) learn logic rules and MFs simultaneously; (4) learn logic rules and MFs sequentially.

In our study an effective fuzzy MFs optimization and rule base extraction and simplification are used simultaneously. To maximize an FLC performance, the construction of an FLC is divided into two phases: (1) an adaptive learning method development for tuning the MFs; (2) an automatic rule learning and reducing mechanism [30].

#### 3.2.1. Encoding Method for Membership Function

Normally three or four parameters are required, respectively, for triangular and trapezoidal MFs, for MF tuning. These parameters are also dependent among themselves for each MF, so additional constraints should be imposed. In case of problems with many variables, tuning MFs leads to optimization with very complex search spaces. Due to this fact, alternative encoding methods have been proposed e.g., Gacto et al. [25] introduced 2-tuple linguistic representation of MFs parameters or very popular using isosceles triangles as MFs, which is encoded by two parameters: centroid and width [30]. These methods of encoding solve the inside dependency problem in MF but the dependency among MFs is limited by additional constraints.

**Figure 2:**
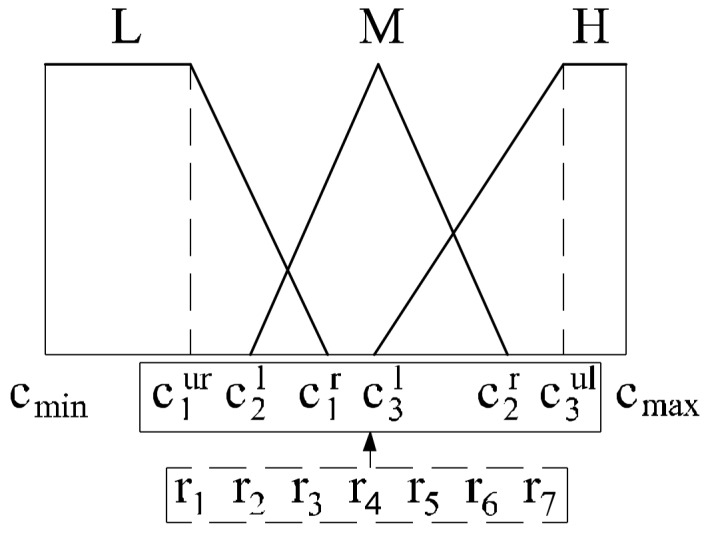
Encoding methods for membership functions (MFs).

In our study the method presented in the article by Chiou and Lan [39] is used. In this encoding method both dependency problems are allowed. Unlike the original work, where encoding was used only for triangular MFs, the methods are expanded to trapezoidal MFs and real coding is applied. The encoding method assumes that the first and last degrees of fuzzy numbers are left- and right-skewed triangles for output values and trapezoids for input values of an FLC. The others are isosceles triangles as shown in Figure 2. The input variable with three linguistic degrees has six parameters to be calibrated and their orders are:

(1) $$c_{\max}=c_{3}^{r}=c_{3}^{\mathrm{ur}}\geq c_{3}^{\mathrm{ul}}\geq c_{2}^{r}\geq \begin{array}{c} c_{3}^{l}\\ c_{1}^{r} \end{array}\geq c_{2}^{l}\geq c_{1}^{\mathrm{ur}}\geq c_{1}^{\mathrm{ul}}=c_{1}^{l}=c_{\min}$$

where $c_{\max},c_{\min}$ are the maximum and minimum values of the variable. To tune these six parameters seven variables $\left(r_{i},i=1,\ldots ,7\right)$ are designed:

(2) $$c_{1}^{\mathrm{ur}}=c_{\min}+r_{1}\cdot \theta ,\;c_{2}^{l}=c_{1}^{\mathrm{ur}}+r_{2}\cdot \theta ,\;c_{1}^{r}=c_{2}^{l}+r_{3}\cdot \theta ,\;c_{3}^{l}=c_{2}^{l}+r_{4}\cdot \theta ,\;c_{2}^{r}=\max\{c_{1}^{r},c_{3}^{l}\}+r_{5}\cdot \theta ,\;c_{3}^{\mathrm{ul}}=c_{2}^{r}+r_{6}\cdot \theta$$

where:

(3) $$\theta =\frac{c_{\max}-c_{\min}}{\sum_{i=1}^{7}r_{i}}$$

Variables $r_{i}$ are coded as real values. The detailed information for triangular MFs (output values) was presented in the article by Chiou and Lan [39].

#### 3.2.2. Encoding Method, Learning and Reduction Technique for Logic Rules

There are many methods which have been developed for fuzzy rule optimization. In our work the method proposed by Shill et al. [30] is adopted. The most important information about this method is presented in the next section.

**Figure 3:**
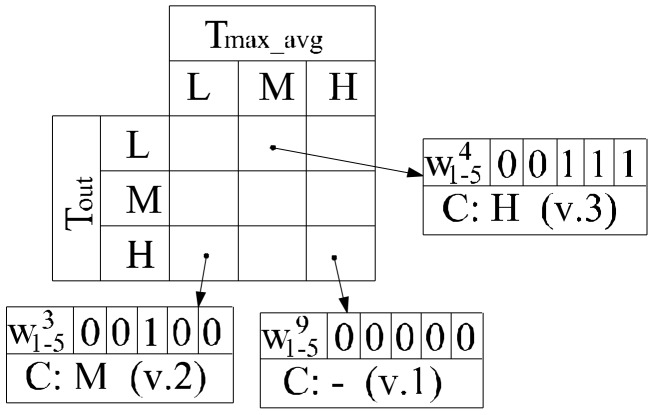
Consequents combining technique.

Original fuzzy rule base is automatically generated in the first step. In the second step redundant, irrelevant and erroneous rules are removed by setting their all consequent weight factors to zero, merging the conflicting rules that have the same antecedents value during the learning process. Figure 3 shows an example of a merging technique of conflicting rules with different consequent values (C in Figure 3) for the first part of the proposed FLC for optimization of ventilation.

Fuzzy control rules are modified by adding a weight factor, $w_{m}^{j}$, to the consequences of each rule, where j is the number of rule (in Figure 3 *j* = 1, …, 9), m is the number of linguistic variable for the output variable, (*m* = 1, …, 5 = : $l_{m}$: $B_{m}=\{\mathrm{VL},L,M,H,\mathrm{VH}\}$). This weight is used to determine whether the rule is included or not (Figure 3), e.g.:

(4) $$R^{3}={\mathrm{If}\;T}_{\max\_\mathrm{avg}}{\;\mathrm{is}\;L\;\mathrm{and}\;T}_{\mathrm{out}}{\;\mathrm{is}\;H\;\mathrm{Then}\;T}_{\mathrm{vent}\;}\;\mathrm{is}\;\overset{5}{\underset{m=1}{\sum }}w_{m}^{3}B_{m}$$

The consequent value of rule is calculated as follows:

v.1: if $\sum_{m=1}^{l_{m}}w_{m}^{j}=0$—exclude the *j*th rule from the candidate rule base,
v.2: if $\sum_{m=1}^{l_{m}}w_{m}^{j}=1$—include the *j*th rule directly to the rule base,
v.3: if $\sum_{m=1}^{l_{m}}w_{m}^{j}>1$—combines the two or more consequences into one using the equation:
  (5) $$\mathrm{NV}=\frac{1}{n}\overset{n}{\underset{i=1}{\sum }}N_{i}$$
  where: n is the number of consequence, $N_{i}$ is the center numeric values of fuzzy rule consequence. A linguistic variable with the highest membership value for NV is the result of different consequences.

Binary encoding for logic rules is used. Each chromosome is a vector of binary numbers whose size is the product of possible rules number and the number of possible consequences for each rule. Therefore a total chromosome is composed of two main sub chromosomes containing: the MFs parameters (real encoding) and the parameters that are used for tuning and reducing rule (binary encoding).

#### 3.2.3. Multi-Objective Optimization Process

Thermal comfort means satisfaction with the thermal environment. The appropriate mathematical models make it possible to evaluate the thermal environment quality and determine the percentage of the dissatisfied with the thermal conditions [40,41,42]. In this study the indicator used to assess thermal comfort is the Predicted Mean Vote (PMV), based on Fanger’s model [43]. Basing on the temperature measurements in a room, the average radiation temperature as well as the airflow rate, the PMV allows to predict the mean thermal sensation of a group of people whose activity is known and whose clothes have the known thermal insulation. The PMV index predicts the average mean score for a large group with the seven-point thermal sensation scale (from −3 (too cold) to +3 (too warm)). Among a population the lowest Percent of People Dissatisfied (PPD) with the thermal environment is when the PMV equals zero. In our study the absolute PMV value of 0.5 is assumed as the limit of the comfort zone, similarly to other works [44]. It is the medium exigent comfort category in PN-EN ISO 7730:2006 [40] (category B from range A–C) and PN-EN 15251:2012 [41] (category II from range I–IV) standards.

It is the aim of an FLC, as described above, to control the supply of ambient airflow in order to improve thermal comfort in rooms. A supplemental airflow will lower too high indoor temperature with a cooler outdoor air. Some undesirable effects may occur in the process: in case of excessive cooling, the heating will turn on and the heat demand will increase. Clearly, such operation of the system will be unfavourable.

Therefore, in order to optimize the FLC, two parameters were considered: E_tot_—energy demand of the building and H_dis_—number of hours with |PMV| > 0.5, representing the cumulative time with discomfort over the period of optimization.

Minimizing the two conflicting parameters ensures that the PMV index improvement in a building will not result in too high energy demand.

Multiobjective Evolutionary Algorithm (MOEA), which is a kind of a GA, enables one to optimize more objectives simultaneously. It is based on Pareto-dominance. Unlike classical weighted-sum approach, MOEA gives more solutions than a single optimization problem. In the research of the built environment Non-dominated Sorting Genetic Algorithm (NSGA-II) [45] is the most widely used. But the work [25] shows that in case of an FLC optimization, Exploration-Exploitation SPEA2 (SPEA2_E/E_) method, which is based on the Strength Pareto Evolutionary Algorithm (SPEA2 [46]) produces better results. This method is applied for the FLC optimization in our study. In the original work this method was used for the optimization of MF and the selection of defined rule for HVAC system. Due to the complexity of the problem which was analysed in this work all objective functions were combined to one. As presented earlier in our paper, this method is also applied for creating and learning a fuzzy logic rule. Therefore some modification of the original method is conducted. In our work Pareto front has been obtained as the final result. In the next section, some basic information about the optimization algorithm is presented.

The previous sections described the structure of the FLC in detail. Each chromosome is composed of two sub chromosomes: 1—vector of real valued numbers for MFs encoding, 2—vector of binary valued numbers for rule encoding. The objective of the optimization process is to learn and reduce the rule base and optimize the MFs parameters. In comparison with SPEA2 methods some mechanisms that give more selective pressure to high performance solution and better exploration was added in SPEA2_E/E_:

*An incest prevention mechanism*: binary tournament selection with replacement is applied for paired parents for mating during the reproduction process. Only those parents whose hamming distance divided by 2 is higher than a difference threshold D (D = L/4, where L is the sub-chromosome 1 length) are crossed [25].

*Crossover*: to maintain the appropriate balance between exploitation and exploration different (during time of simulation) crossover operator is applied in our work. In the first half of simulation steps only one child is a result of crossover of two parents. At this stage a highly disruptive crossover HUX and BLX-05 operators are used for the binary and real part of chromosome respectively. These operators replace classical mutation and crossover in our study. In the second half of the simulation steps parents have two children. The same operators as in the first stage of the simulation are used for creating the first of them. Only the binary part is different for children. As in [25] Euclidean distances are computed between child and parents real part of chromosomes. The parent rule base with the closest distance to the offspring is copied directly to the second sub-chromosome of the second child. It increases the exploitation on the second part of simulation.

*A restart process*: this substitutes the usual GAs mutation. It is only applied when the population has converged. The same condition, as in [25], is used to initiate the restart process. In our work, a quarter of population is created as a copy of chromosomes from Pareto zone to protect the chromosomes with high performance. The remaining are generated as mutated elements of Pareto zone with 0.35 mutation probability.

### 3.3. Employed Tools

As shown in other studies, various methods are used to calculate the energy demand in buildings [47]. Programs such as ESP-r, TRNSYS, EnergyPlus, IDA-ICE are commonly used for building performance simulation (BPS). However, the use of BPS programs requires the building of detailed models [48,49,50]. As a results, not only do we receive global results, but also detailed ones for individual zones. Other methods are also used: statistical methods and methods based on artificial neural networks. These methods, which operate as black box models and base on the experimental data, have been strongly developed in recent years [51,52]. Simulations are then carried out much faster, but usually the whole building is treated as one zone and we only get global results. Often, they make it impossible to take the full dynamics of the building into account [53]. To estimate thermal comfort in this work, we need the results to be divided into individual rooms (a multizone model), taking into account the dynamics of internal and external thermal loads throughout the year. Therefore, the EnergyPlus [54] program was used for calculations. The MOEA optimization algorithm defined in the previous section is implemented in MATLAB (R2017a, The MathWorks Inc., Natick, MA, USA) and it is automatically coupling with energy simulation tool. The energy modelling tool EnergyPlus, which allows integrated calculations of the transfer of mass and energy inside the building, was used for the simulation and calculation of the objective function: H_dis_, E_tot_. It is clear from the previous sections that input and output data of the FLC change dynamically during the simulation time. Therefore co-simulation between these programs has to be implemented. The co-simulation approach represents a particular case of simulation scenario where at least two simulator solve the coupled differential-algebraic systems of equations and exchange the data that couple these equations during the time of integration [55]. The MLE+ [56] MATLAB toolbox is used for co-simulation between MATLAB and EnergyPlus. Fuzzy Logic Toolbox is used for the execution of all interior operations in the FLC, for data (MFs parameters, rule base) which is received from the GA. To the authors’ knowledge this is the first time where optimization process of an FLC has been performed as an all-year co-simulation with the connection between the programs that was proposed here. The final goal of the optimization is the Pareto front, which represents the set of non-dominated solutions. The last step is to select a solution that represents the best FLC configuration. This process is called Multi-Critera Decision Making (MCDM).

Different criteria can adopt for the MCDM. In our study the two methods are adopted:

- the utopia point method (utopia criterion (UC)): the best FLC is the closest to the ideal point (point that minimizes both objective function). As stated in [57] this approach has already been used in many engineering applications;
- the maximum thermal comfort method (thermal comfort criterion (TCC)): H_dis_ for this FLC is minimal. Due to the character of this work, the method is used and compared with the utopia point method.

**Figure 4:**
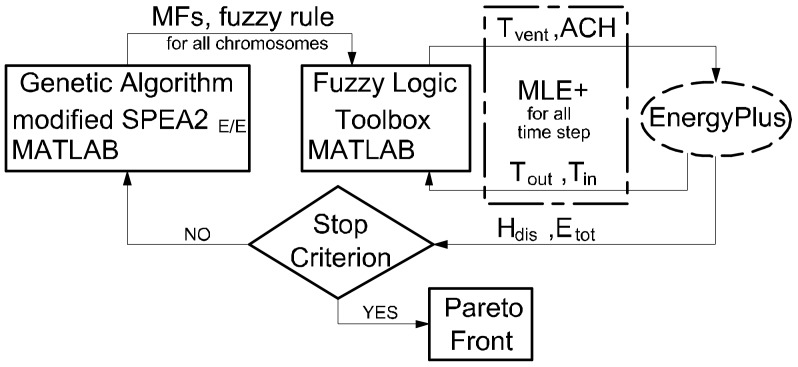
Flowchart diagram for the developed simulation/optimization tool.

Figure 4 shows the structure of the simulation and optimization environment.

### 3.4. Research Building Description and Energy Simulation Assumption

**Figure 5:**
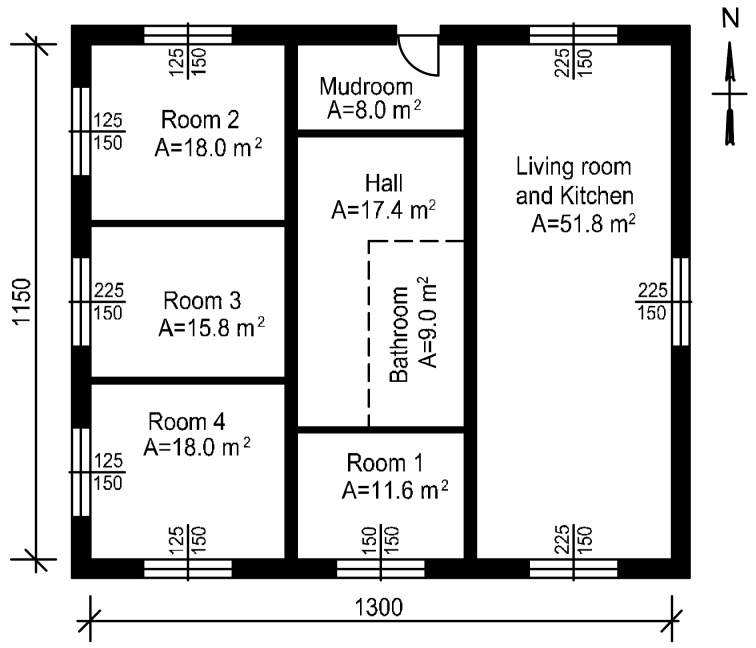
Ground floor view.

A single-family detached house without a cellar and with an unusable attic was chosen for the research. The total area of rooms is 150 m^2^ and the height of rooms is 2.6 m. The ground floor of the building is shown in Figure 5. The walls are of brick construction with polystyrene insulation (*U* = 0.22 W/m^2^K), the ceiling is a ferroconcrete structure with mineral wool insulation (*U* = 0.18 W/m^2^K) and the roof is covered with ceramic tiles and is uninsulated. The heat transfer coefficients (*U*) of the external partitions are according to the Polish requirements for thermal insulation for newly designed buildings [58]. The windows area amounts to 23.3 m^2^ (including glazing 15.7 m^2^). The solar heat gain coefficient of window glazing is 0.49, visible transmittance is 0.72 and heat transfer coefficient is 1.0 W/m^2^K.

The simulations were performed on a multizone model with 15-minute time step using the reference weather data for Katowice [59]. Internal heat gains were introduced into model: four occupants with the activity level of 1.2 met, two computers, a TV set and kitchen equipment (heat gains values according to the literature [60,61]) and lighting (10 W/m^2^). An hourly schedule for the presence of occupants and for the use of lighting and equipment was adopted in each room. The detailed schedule of internal heat gains is presented in [24] and it is assumed that at least one person stays at home all the time.

A minimum ventilation airflow was assumed according to the Polish standard PN-83/B-03430/Az3:2000 [62]. For the analysed house such a minimum airflow is 120 m^3^/h (it is about 0.3 air change per hour). A heating and cooling set point was assumed according to the PN-EN 15251:2012 standard [41]. The recommended design values of the minimum and maximum indoor operative temperature amount to 21.0 °C and 25.5 °C. The paper by Kaczmarczyk et al. [63] showed that in a standard room in Polish climate, the operative temperature diverges from air temperature about 0.5–1.0 K. In winter air temperature is higher than operative temperature. In summer period the situation is reversed. Therefore in our study we assume a heating set point for air temperature of 22.0 ℃, but between 10 pm and 5 am night time decreases of 3 K have been assumed. The cooling set point for air temperature is assumed for all day at the level of 25.0 °C. The ideal control for heating and cooling was assumed. The PMV values are also calculated by EnergyPlus, using a constant air velocity of 0.1 m/s in winter, 0.2 m/s in summer and 0.15 m/s during the rest of the year. Clothing factor amounts to 1.0 clo when outdoor temperature is below 14 °C, 0.5 clo for temperature above 22 °C and 0.8 clo during the rest of the year.

### 3.5. Thermal Model Validation

Prior to the main research (optimization), a numerical verification of the constructed thermal model was performed. The hourly and the annual heating and cooling demand for the building were verified. The results obtained in EnergyPlus program for the building (with constant airflow) were compared to the results obtained in an alternative energy simulation program: ESP-r [64]. The ESP-r software and energy multizone models were repeatedly validated with experimental data by the authors [49,50]. The correlation coefficient of hourly values is 0.99 and the difference in annual energy demand (heating + cooling) calculated in both programs is 8%. The hourly and daily values of the energy demand obtained from EnergyPlus were mostly higher, however similar results were obtained in others studies [65]. The detailed model validation is presented in [24]. Based on this analysis it can be stated that the presented model is characterized by sufficient accuracy and can be used for thermal calculations of the building.

## 4. Results

The values of the parameters used in MOEA are: 300 evaluation, population size of 60, external size of 16, 0.35 mutation probability. After the preliminary simulations, it was noticed that such parameters are a compromise between the duration of the simulation and the improvement in the obtained results. Co-simulation between MATLAB and EnergyPlus programs is time consuming and takes 11 h for the assumed parameters. The simulation was carried out three times. The best results (minimal H_dis_) have been shown below. The FLC was optimized for two different cases.

The optimized results are to be compared to two base systems:

- a building with ventilation using ambient air maintained at a constant level 0.5 h^−1^ from 1st May to 30th September and on the level 0.3 h^−1^ in the rest of the year (case B1);
- a building with ventilation using ambient air maintained at a constant level 0.3 h^−1^ during the whole year and air conditioning system (case B2).

**Figure 6:**
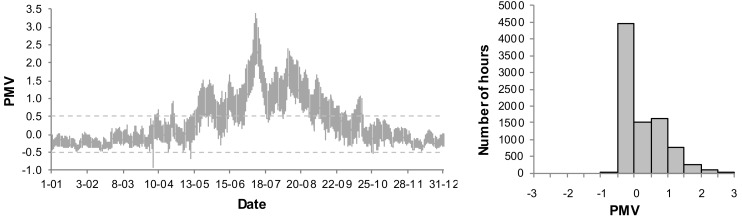
Annual variation and frequency distribution of Predicited Mean Vote (PMV) for living room in B1 system.

**Figure 7:**
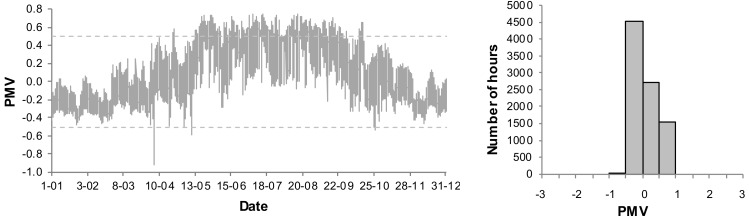
Annual variation and frequency distribution of (Predicited Mean Vote) PMV for living room in B2 system.

The presented analyses do not include the PMV < −0.5, when the night temperature decreases. An annual variation of the PMV for the living room and frequency distribution of the PMV values for the basic systems B1 and B2 are presented in Figure 6 and Figure 7. As the living room is connected to the kitchen, the thermal conditions are subject to greatest fluctuations. Thus the thermal conditions in that room are expected to be the worst.

In case B1, when the ventilation is constant on the level 0.5 h^−1^, very high values of the PMV index occur in the summer. Additionally, the index is too high in transitional periods (spring, autumn). 17.9% of the time the conditions do not meet the assumed criteria and the PMV reaches its maximum value. It is, however, an extreme case with no supplemental ventilation. In case of B2, with the assumed coefficients (air velocity, clothing factor) the PMV index exceeds the set point of 0.5. Its maximum value, however, is much lower than in case of B1 and amounts to 0.8. In case of B2 we may lower a cooling set point in order to improve thermal comfort. It will, however, result in higher energy demand.

Case 1: FLC optimization for a building with ventilative cooling

**Figure 8:**
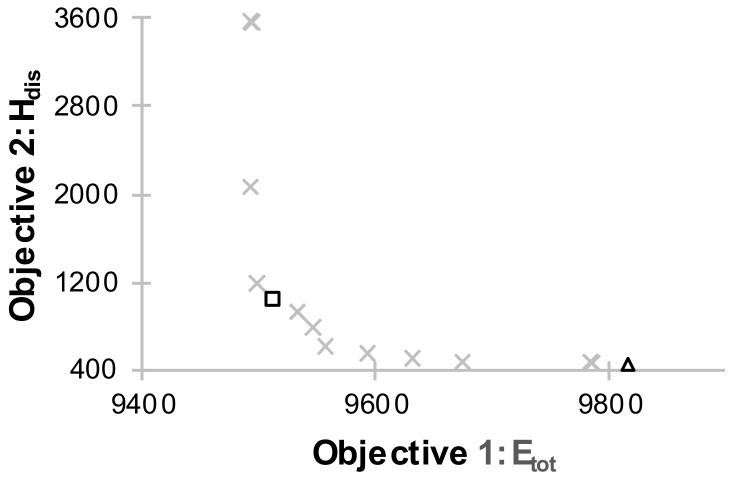
Pareto front for case 1.

**Figure 9:**
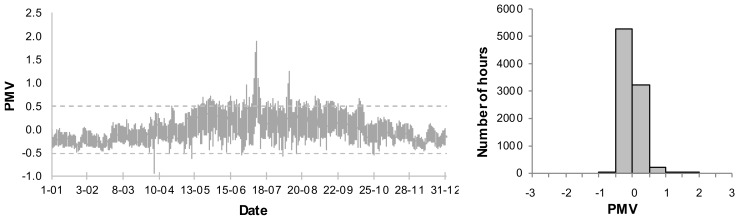
Annual variation and frequency distribution of PMV for living room in case 1.

**Figure 10:**
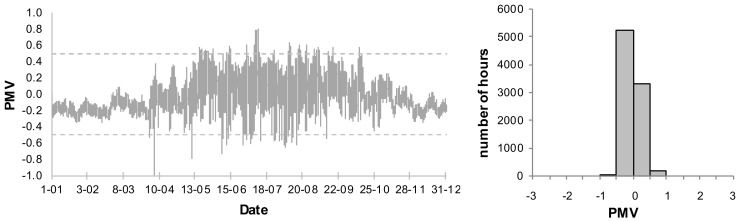
Annual variation and frequency distribution of PMV for room 1 in case 1.

In the first case the operation of mechanical ventilation in the building was optimized. The Pareto front, which shows the non-dominated configurations of the FLC is presented in Figure 8. The points marked with a square and a triangle are the selected solutions based on the criteria: UC and TCC, respectively. The annual variation and frequency distribution of the PMV values for the FLC that was selected based on TCC have been presented in Figure 9 for the living room and in Figure 10 for room 1. Table 1 shows the global results for the whole building for base and optimized systems.

As shown in Table 1 an appropriate control of supply airflow can significantly improve the thermal comfort of residents. H_dis_ is 1% and 2.3% respectively for the TCC and UC. For TCC H_dis_ is shown in Table 2 for individual rooms.

The living room, connected to the kitchen and with a rotating schedule of occupants, has the highest H_dis_, but the value does not exceed 2%. The maximum PMV index in the living room is twice as high as in room 1 (Table 1, Figure 10). Thanks to the appropriate control of mechanical ventilation, good thermal comfort may be achieved in case of the rooms where the internal gains are low (room 1: lighting, computer, one person). The analysis of the results above shows that the optimum supply ambient airflow in transitional climate ensures thermal comfort. The living room is the only one which might require supplemental mechanical cooling system. It is essential that the H_dis_ decrease resulted in a slight increase in energy demand. For TCC and UC it is 3.6% and 0.4% respectively when compared to B1 (Table 1). The maximum PMV value also decreased and did not exceed 2.0. However, the value is still high. It is possible to lower the value only by using mechanical cooling. When using passive ventilative cooling we have to accept that the temperature values in rooms in the hottest periods will be too high. Even the air change rate, above 7 h^−1^ will not prevent the building from overheating in the hottest days. Yet such cases are occasional.

**Figure 11:**
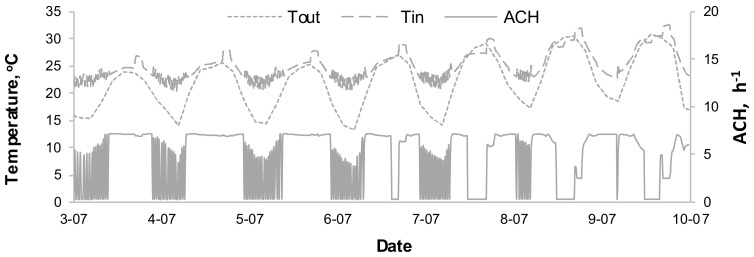
Variation of T_out_, T_in_ and ACH in living room during one chosen week.

Figure 11 presents the temperature variation (outdoor and indoor) in the living room as well as air change rate in the hottest week of the year using TCC controller. The rapid increases in temperature in the Figure overlap with the operations of an oven in the kitchen. When the outdoor temperature exceeds the indoor one, the ACH is at the minimum value of 0.3 h^−1^. Since both heating and cooling have been designed as perfectly tracked, the ACH changes in case 1 were not limited in any way in consecutive time steps. Hence the dramatic ACH changes in the graph, which influence indoor temperature, fluctuations, even during the night hours. This is due to the initial assumptions of the ideal controller for HVAC systems.

The H_dis_ in the building where the supply ambient airflow is optimized is lower than in the building with mechanical cooling (B2), which might be surprising. Considering H_dis_ only, mechanical ventilation with the optimized controller produces better results than the non-controlled cooling system.

**Figure 12:**
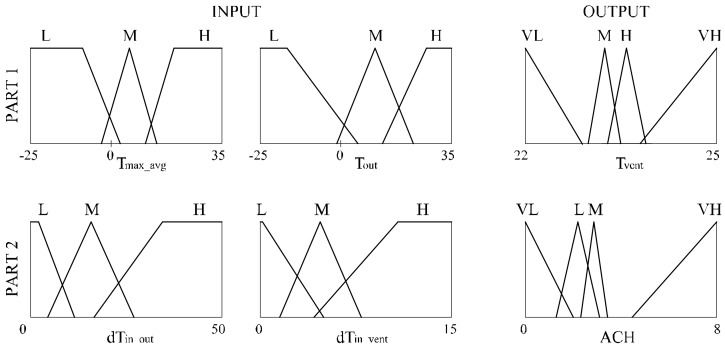
Tuned MF for case O1-TCC.

**Figure 13:**
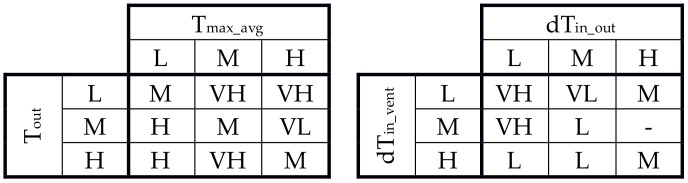
Rule base for O1-TCC.

In Figure 12 and Figure 13 the final MFs and rule base of the FLC for the case 1-TCC is presented. Only one rule has been reduced in fuzzy rule base. Moreover, in the optimization process two MFs have been removed. As it has been mentioned before, five MFs for outputs values (VL, L, M, H, VH) were assumed for the initial FLC. In the optimized FLC two of them (L—for first part, H-for second part) were not chosen for the rule base (Figure 13). This MFs was also deleted in Figure 12. Reducing the rule and the MFs improves the system readability. The number of reductions was similar in case of every analysed controller.

In addition, a simulation with the rules optimization only was carried out. It was assumed that the adjustment functions are evenly distributed. In this case, E_tot_ = 9572 kWh and H_dis_ = 7706 h (which is 18% of the year) was obtained for UC. Comparing these results with the results for the 1-UC case, one can notice a minimal increase in the heat demand and a significant increase in H_dis_ (for the case of 1-UC H_dis_ = 2.3%). It can therefore be concluded that the MF optimization improves the obtained results.

Case 2: FLC optimization for building with ventilative cooling and mechanical cooling

**Figure 14:**
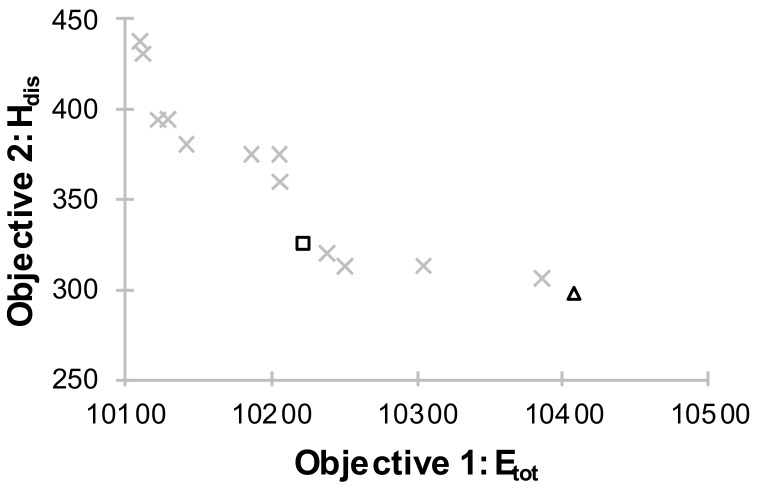
Pareto front for case 2.

**Figure 15:**
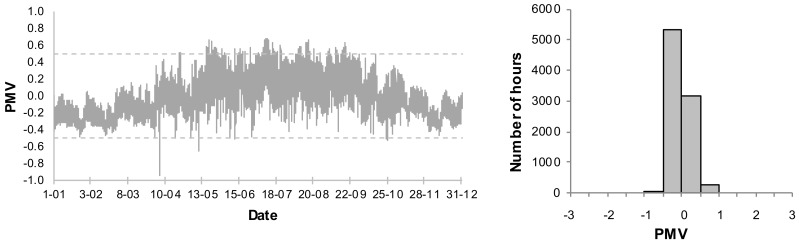
Annual variation and frequency distribution of Predicited Mean Vote (PMV) for living room in case 2.

In Case 2 the operation of mechanical ventilation, which has an auxiliary role for the air conditioning system, was optimized. Such a system should be characterized by a lower energy demand and better thermal comfort. Mechanical cooling system works in the same way as in B2. The Pareto front is presented in Figure 14. The annual variation and frequency distribution of the PMV values in the living room for the FLC that was chosen basing on the TCC have been presented in Figure 15.

An air conditioning system combined with ventilative cooling gives better results (Table 1). Slight savings in energy demand (maximum of 6% for the case 2-UC controller compared to B2) were achieved. The H_dis_ decreased dramatically, amounting to 0.7% for case 2 and 8.7% for case B2. Additionally, the maximum PMV (max PMV = 0.7) is slightly lower. Such a low value is possible only for the systems with mechanical cooling, which are at the same time the most expensive ones (the paper does not analyse this aspect). The comparison of cases 1 and 2 shows that the energy demand in both cases differs only slightly. Heat demand is much bigger than the cooling demand in Polish climate (e.g., for case 2-UC the heat demand = 9712 kWh, cooling demand = 511 kWh). In such a system cooling will turn on during the hottest days. Cooling demand is three times lower than the base B2 model.

## 5. Conclusions

The paper examines the possibilities to improve thermal comfort in detached houses in a transitional climate (in Poland) using mechanical ventilation. A new fuzzy logic controller, which will operate the mechanical ventilation to minimize the number of hours with poor thermal comfort, has been proposed. PMV measures the thermal comfort for the purpose of this work. The modified MOEA, based on SPEA2 and SPEA2_E/E_, has been used to optimize the MF as well as the rule base of a fuzzy logic controller. The proposed MOEA has been implemented in MATLAB and coupled with EnergyPlus using MLE+. The co-simulation to optimize the FLC with the use of the above-mentioned programmes has been carried out for the first time. The possibilities to support mechanical cooling by mechanical ventilation have also been examined.

The simulation results for the detached house demonstrated great potential to improve thermal comfort using optimally controlled mechanical ventilation. The number of hours when the assumed thermal comfort range (−0.5 < PMV < 0.5) exceeds the value is only 1%. Applying mechanical ventilation only with the use of ambient air, we have to accept a high PMV of 2.0 in rooms with high internal gains in hot periods. Such cases, however, are very uncommon. The simulations show that expensive cooling systems are unnecessary in detached houses in transitional climate, but if such costly systems are applied to improve the thermal comfort of the occupants, the mechanical ventilation using ambient cool air should support them in order to reduce energy demand.

In our work the cooling and heating demand of the zone was achieved by using an ideal air system in EnergyPlus. Hence, no restrictions on the stability of the fans have been introduced. In further work we will strive for a more accurate modelling of ventilation devices. Also, introducing the third objective function could be a good method of improving the system stability.

## Figures and Tables

**Table 1:** Results for all analysed cases.

Case	MCDM	H_dis_, %	E_tot_, kWh	E_cool_, kWh	max PMV	max/avg ACH, h^−1^
B1	-	17.9	9478	-	3.4	0.5/0.38
B2	-	8.7	10891	1462	0.8	0.3/0.3
1	TCC	1.0	9817	-	1.9	7.2/0.82
1	UC	2.3	9514	-	2.0	7.1/0.70
2	TCC	0.7	10514	506	0.7	7.2/0.69
2	UC	0.7	10223	511	0.7	7.0/0.66

MCDM: Multi-Critera Decision Making; PMV: Predicited Mean Vote; ACH: air change rate; TCC: thermal comfort criterion; UC: utopia criterion.

**Table 2:** H_dis_ in case 1-TCC.

Type of Room	H_dis_, %
Living room	1.92
Room 1	0.81
Room 2	0.90
Room 3	0.96
Room 4	0.63
